# RNA-sequencing transcriptome analysis of *Avicennia marina* (Forsk.) Vierh. leaf epidermis defines tissue-specific transcriptional response to salinity treatment

**DOI:** 10.1038/s41598-023-34095-x

**Published:** 2023-05-10

**Authors:** Huan Li, Chao-Tian Lv, Yun-Tao Li, Guo-Yv Gao, Ya-Fei Meng, Yv-Le You, Qi Tian, Kun-Qi Liang, Yu Chen, Hao Chen, Chao Xia, Xiang-Yun Rui, Hai-Lei Zheng, Ming-Yue Wei

**Affiliations:** 1 College of Food and Bio-Engineering, Bengbu University, Bengbu, Anhui 233030 People’s Republic of China; 2 School of Ecology, Resources and Environment, Dezhou University, DeZhou, Shandong 253000 People’s Republic of China; 3 Key Laboratory for Subtropical Wetland Ecosystem Research of MOE, College of the Environment and Ecology, Xiamen University, Xiamen, Fujian 361005 People’s Republic of China

**Keywords:** Plant sciences, Plant stress responses

## Abstract

*Avicennia marina* (Forsk.) Vierh. is a typical mangrove plant. Its epidermis contains salt glands, which can secrete excess salts onto the leaf surfaces, improving the salt tolerance of the plants. However, knowledge on the epidermis-specific transcriptional responses of *A. marina* to salinity treatment is lacking. Thus, physiological and transcriptomic techniques were applied to unravel the salt tolerance mechanism of *A. marina*. Our results showed that 400 mM NaCl significantly reduced the plant height, leaf area, leaf biomass and photosynthesis of *A. marina*. In addition, 1565 differentially expressed genes were identified, of which 634 and 931 were up- and down-regulated. Based on Kyoto Encyclopedia of Genes and Genomes metabolic pathway enrichment analysis, we demonstrated that decreased gene expression, especially that of *OEE1*, *PQL2*, *FDX3*, *ATPC*, *GAPDH*, *PRK*, *FBP* and *RPE*, could explain the inhibited photosynthesis caused by salt treatment. Furthermore, the ability of *A. marina* to cope with 400 mM NaCl treatment was dependent on appropriate hormone signalling and potential sulfur-containing metabolites, such as hydrogen sulfide and cysteine biosynthesis. Overall, the present study provides a theoretical basis for the adaption of *A. marina* to saline habitats and a reference for studying the salt tolerance mechanism of other mangrove plants.

## Introduction

Mangroves, which occupy ocean coastlines throughout the tropics, are among the most productive ecosystems worldwide^1^. It has been reported that multienvironment factors, such as temperature, tide level, and water salinity, impact the survival rate of mangrove plantations. Among these factors, high salinity is a characteristic of the coastal intertidal zone habitats, and a major factor restricting the growth of mangrove seedlings^2^.

Generally, salinity stress first damages the osmotic equilibrium and ion balance, and then causes oxidative stress, resulting in variation in membrane permeability, disorder of material and energy metabolism, and accumulation of toxic substances; thus, the growth and development of plants are affected^3^. As typical halophytes, mangroves can be divided into the following types: nonsalt-secreting and salt-secreting plants. Mangroves have formed sets of unique characteristics to adapt well to high salinity conditions^4^. It is generally accepted that nonsalt-secreting mangrove plants are capable of extruding excess salt through ultrafiltration at the roots^5^. In contrast, the most unique feature of salt-secreting mangrove plants is the development of salt glands which can effectively reduce the concentration of Na^+^ in plant leaves. Under high salinity, a high K^+^/Na^+^ ratio in the cytoplasm is essential for maintaining the normal metabolic activity of cells. It has been reported that mangroves can regulate nonselective cation channels to limit the transport of Na^+^ or partition excess ions into vacuoles; this is achieved through Na^+^/H^+^ reverse transporters to maintain the optimum K^+^/Na^+^ ratio in cells^6^. In addition, the tolerance of mangrove plants to high-salinity surroundings is closely correlated with the modulation of the expression of certain salt-related genes. Previous studies revealed that the expression of plasma membrane H^+^-ATPase (HA1), betaine/proline transporter (AMT1, AMT2 and AMT3), plasma membrane Na^+^/H^+^ antiporter (SOS1) and vacuolar Na^+^/H^+^ antiporter (NHX1) genes was induced in the salt-treated *Avicennia marina* (Forsk.) Vierh. leaves^6,7^. Recently, Guo et al.^8^ studied the complex response of aquaporins (AQPs) to multiple environmental factors involving salinity, suggesting the essential role of *Kandelia obovata* in adaptation to coastal saline conditions. In addition, the accumulated organic osmotic substances (e.g., hydroxyl compounds, free amino acids, and polysaccharides) and quickly activated antioxidant defence system could maintain the osmotic balance to mitigate the damage of oxidative stress^9^. For example, after salt treatment, the overgeneration of superoxide (O_2_^.–^) in *Bruguiera parviflora* is counterbalanced by enhancing superoxide dismutase (SOD) activity^10^. The activities of SOD and catalase (CAT) were immediately increased after *Bruguiera gymnorrhiza* was transferred from low to high salinity^11^. Although great progress has been made regarding the salt tolerance of mangrove plants, the specific mechanism of salt secretion still needs to be further investigated.

Recently, omics approaches have been increasingly applied to study the environmental adaptability of mangrove plants and their response to biological and abiotic stresses. Zhu et al.^12^ revealed that proteins related to photosynthesis, antioxidation, protein folding and degradation, and cell organization are crucial for the salt tolerance of *Bruguiera gymnorrhiza* under severe salt stress. Wang et al.^13^ employed iTRAQ proteome technology coupled with the transcriptome sequencing method to demonstrate the salt-tolerance regulation of *Kandelia candel* at the protein and mRNA levels. In addition, relevant transcriptome studies have been performed with mangrove plants, such as *A. marina*^14^, *Ceriops tagal* and *C. zippeliana*^15^, *Sonneratia alba*^16^ and *Acanthus ilicifolius*^17^. These transcriptome data provide valuable biological information resources for investigating the origin, evolution, and intertidal ecological adaptation mechanism of mangrove plants. Despite years of research, the tissue-specific transcriptome of mangrove plant responses to salt remains worthy of further study.

*A. marina*, as a pioneer tree species of the mangrove ecosystem and a typical salt-secreting mangrove plant, shows great advantages in salt resistance. Our previous studies showed that the salt crystals on the leaf surface of *A. marina* increased significantly after 400 mM NaCl treatment^4,6^. Further proteomic analysis revealed that the abundances of proteins related to photosynthesis were sharply decreased, and the stomatal and nonstomatal limitations could account for the photosynthetic reduction^2^. However, the mechanism by which salinity treatment regulates transcriptomic profiling in leaf epidermis is not well known for this mangrove species; thus, RNA-Seq technology together with physiological analysis was performed in this study.

## Results

### Effects of NaCl treatment on the growth of *A. marina* seedlings

**Figure 1:**
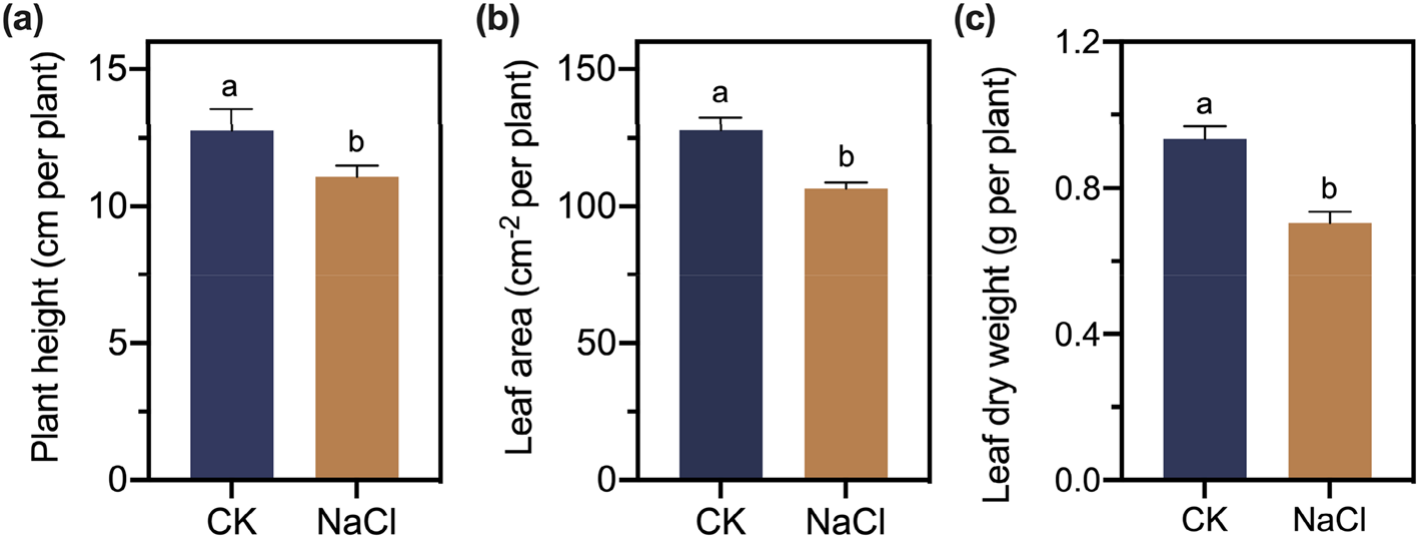
Effect of 0 mM NaCl (CK) and 400 mM NaCl (NaCl) treatment on the plant weight (**a**), leaf area (**b**), and leaf dry weight (**c**). Different letters in each column indicate significant differences with *p* < 0.05.

To study the influence of salinity treatment on the growth of *A. marina* seedlings, several parameters, including plant height, leaf surface area and leaf biomass, were determined under 0 mM NaCl (CK) and 400 mM NaCl (NaCl) (Fig. 1). The results showed that after one month of salinity treatment, the plant heights and leaf areas of *A. marina* seedlings were significantly inhibited. In addition, the leaf biomasses decreased by 24.5% compared to that of the control plants.

### Effects of NaCl treatment on the photosynthetic capability of *A. marina* seedlings

**Figure 2:**
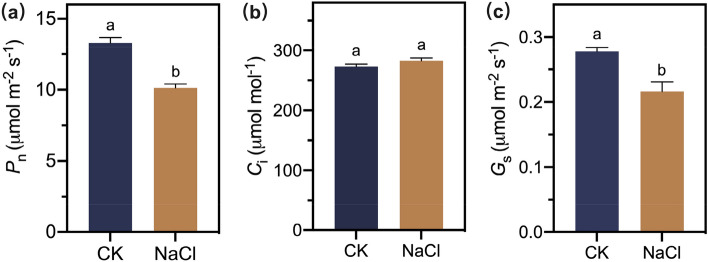
Effect of 0 mM NaCl (CK) and 400 mM NaCl (NaCl) treatment on *P*_n_ (**a**), *G*_s_ (**b**) and *C*_i_ (**c**). Different letters in each column indicate significant differences with *p* < 0.05.

To further study the physiological effects of NaCl treatment on *A. marina* leaves, the photosynthetic capability was thus measured. The results showed that the photosynthetic rate (*P*_n_) and stomatal conductance (*G*_s_) were dramatically lower in the NaCl treatment than that in the CK, while the intercellular CO_2_ concentration (*C*_i_) was not significantly altered (Fig. 2).

### Assembly and functional annotation of transcriptome data in leaf epidermis of *A. marina* seedlings

The transcriptome results are shown in Table 1. A total of 45,312,506–47,111,632 raw reads were detected. After filtration, 37,687,002–45,354,800 clean reads were obtained. The Q20 and Q30 values ranged from 97.88 to 98.64% and 93.60 to 95.64%, respectively. In addition, the GC contents were between 44.81 and 47.34%.

**Table 1:** Summary of assembly results of 0 mM NaCl (CK1–3) and 400 mM NaCl (NaCl1–3) treated *A. marina seedlings*.

Sample	Raw reads	Clean reads	Clean bases	Error (%)	Q20 (%)	Q30 (%)	GC (%)
CK1	47,111,632	45,354,800	6.80 G	0.03	97.88	93.60	45.67
CK2	42,521,038	41,760,254	6.26 G	0.02	98.24	94.65	44.81
CK3	42,912,590	39,835,844	5.98 G	0.02	98.37	95.05	47.34
NaCl1	45,312,506	41,977,602	6.30 G	0.02	98.19	94.57	45.66
NaCl2	45,829,168	37,687,002	5.65 G	0.02	98.64	95.64	46.82
NaCl3	42,980,148	40,348,190	6.05 G	0.02	98.20	94.65	46.24

### Screening of DEGs and analysis of GO and KEGG pathway enrichment

**Figure 3:**
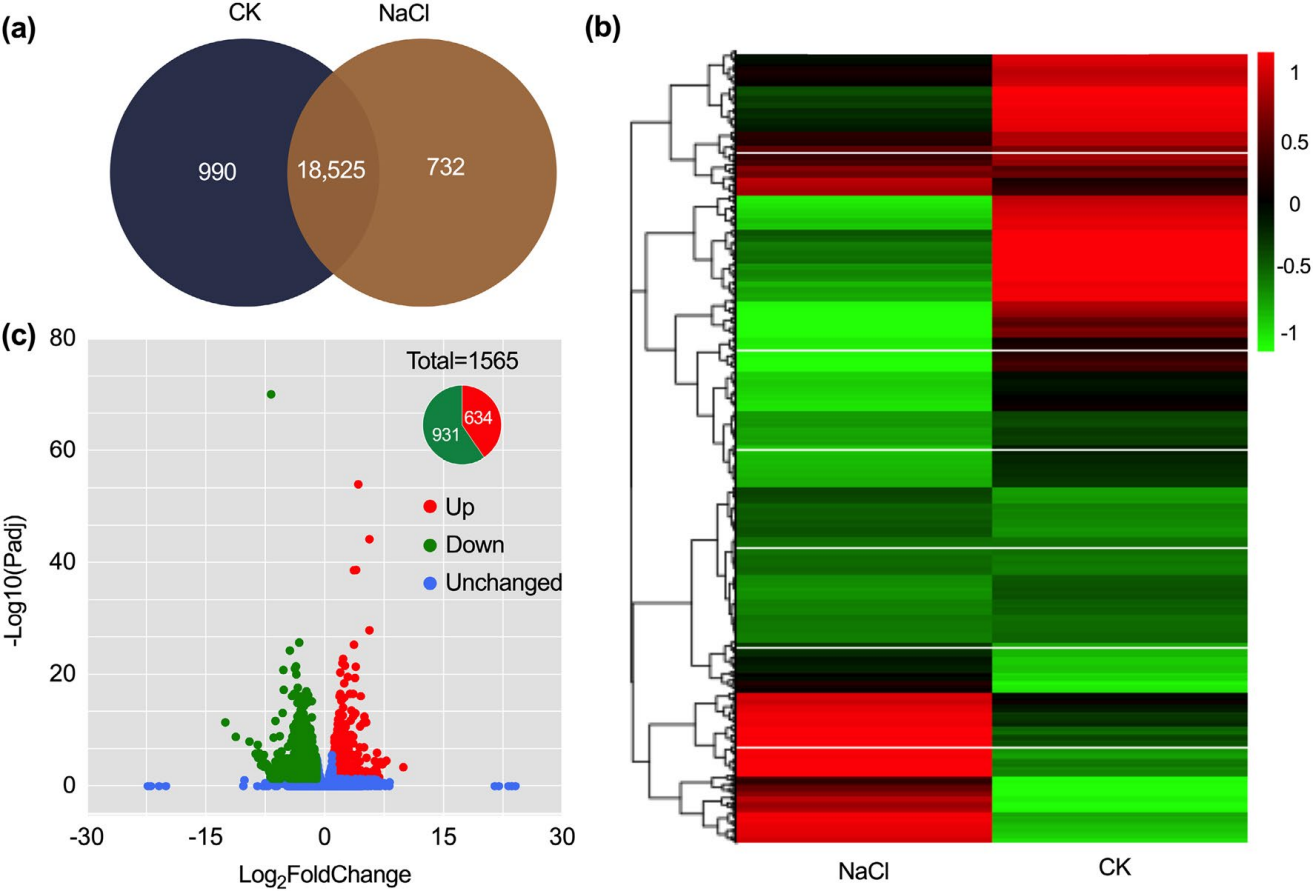
Pictorial representation of the unigenes identified between CK and 400 mM NaCl-treated *A. marina* leaf epidermis. Venn diagram (**a**) and hierarchical clustering (**b**) of unigenes, and the volcanic plot (**c**) representing differential expression profiling.

Then, we conducted hierarchical clustering and comparative analysis of unigenes obtained from the CK and NaCl treatment groups within the Venn diagram and heatmap (Fig. 3a,b). The results showed that 732 unigenes were detected only in the NaCl treated group (Fig. 3b). In addition, 1,565 unigenes were differentially expressed, of which 634 were up-regulated and 931 were down-regulated (Fig. 3c).

**Figure 4:**
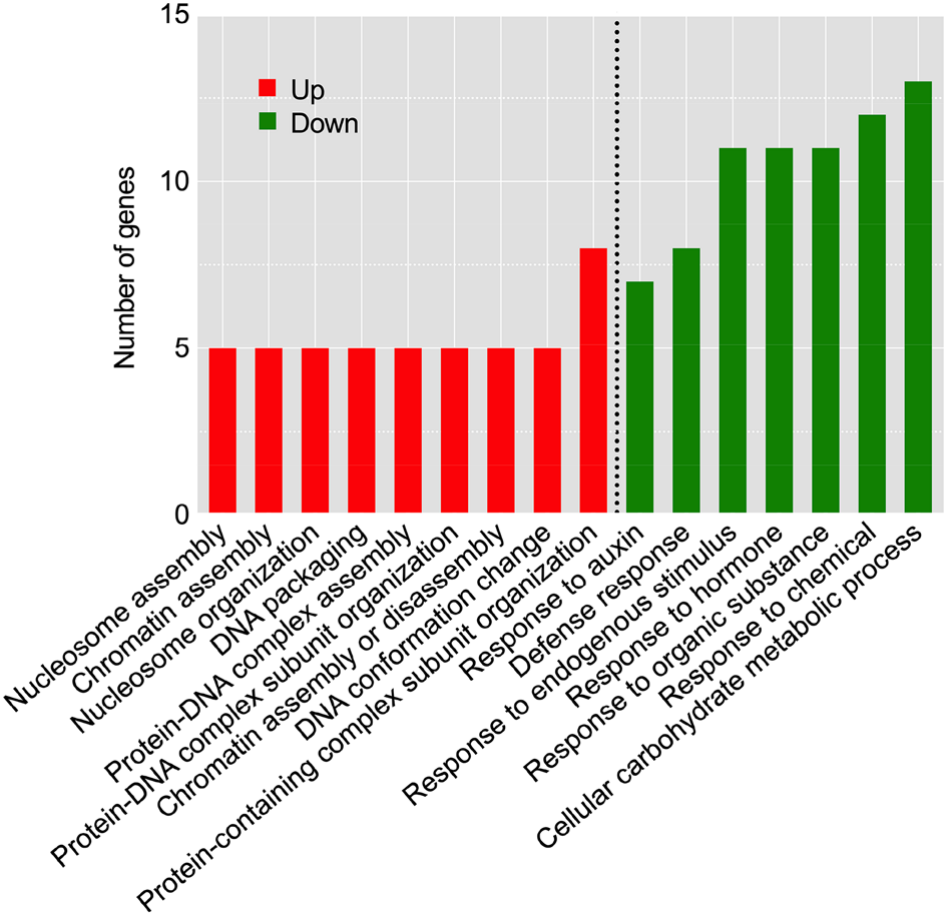
The significantly enriched GO terms of DEGs based on the biological process after 400 mM NaCl treatment. GO terms of up- and down-regulated DEGs are represented in red and green, respectively.

GO enrichment analysis showed that the down-regulated DEGs were mainly concentrated in cellular carbohydrate metabolic process (GO:0044262), response to chemical (GO:0042221), response to hormone (GO:0009725), response to endogenous stimulus (GO:0009719) and response to organic substance (GO:0010033) (Fig. 4). Up-regulation genes were mainly concentrated in protein-containing complex subunit organization (GO:0043933) (Fig. 4).

**Figure 5:**
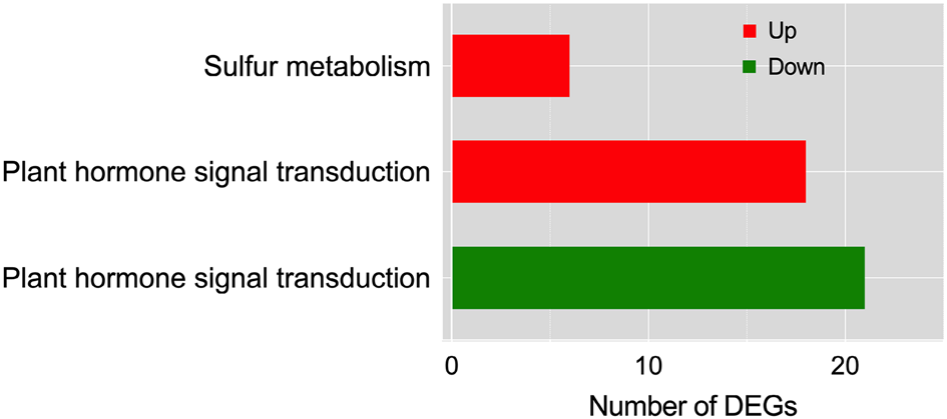
KEGG enrichment analysis of DEGs after 400 mM NaCl treatment. KEGG pathways of up- and down-regulated DEGs are represented in red and green, respectively.

Further enrichment analysis of KEGG metabolic pathways showed that both up- and down-regulated DEGs were dramatically enriched into plant hormone signal transduction (ath04075) (Fig. 5). In addition, the up-regulated DEGs were also enriched in sulfur metabolism (ath00920).

## Discussion

### Effects of NaCl treatment on the photosynthesis of *A. marina* seedlings

Salt stress is among the major abiotic stresses worldwide. The inhibition of plant growth caused by high salt stress is usually manifests as stunted plant growth and inhibited growth and differentiation of plant tissues and organs^18^. The growth of maize leaves was inhibited with the addition of 100 mM NaCl, which was more pronounced in salt-sensitive varieties^19^. Mangrove plants have developed a set of unique woody salt tolerance mechanisms through long-term evolution. Evidence from field observations and in-door experimental studies has shown that mangrove productivity and growth potential are maximized around optimal salinity conditions; however, with high salinity or no salt, a negative effect was observed^2,6,12^. Our previous studies showed that under 400 mM NaCl treatment, large numbers of salt crystals appeared on the leaf surface and a higher rate of Na^+^ efflux from the salt glands of *A. marina* seedlings^4,6^. Our current study further showed that 400 mM NaCl treatment could sharply inhibit the plant height, leaf area and leaf dry weight of *A. marina* seedlings. Similar results were also found for other mangrove plants, such as *Kandelia candel*^13^ and *Bruguiera gymnorrhiza*^12^.

**Figure 6:**
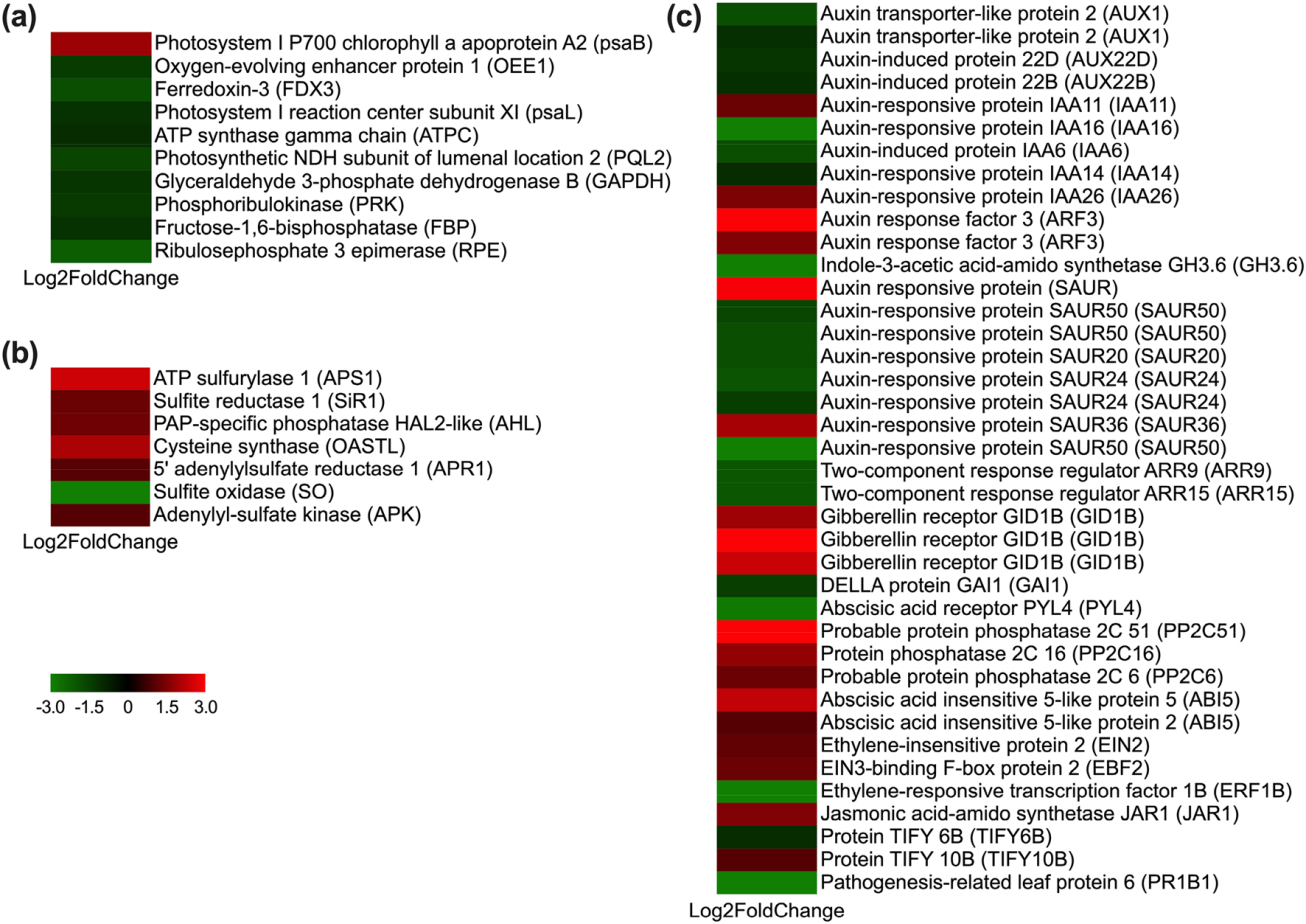
DEGs related to photosynthesis (**a**), hormone signalling (**b**) and sulfur metabolism (**c**). The up- and down-regulated DEGs are represented in red and green, respectively. The colour bar in the lower left indicates the intensity of gene expression profiling.

**Figure 7:**
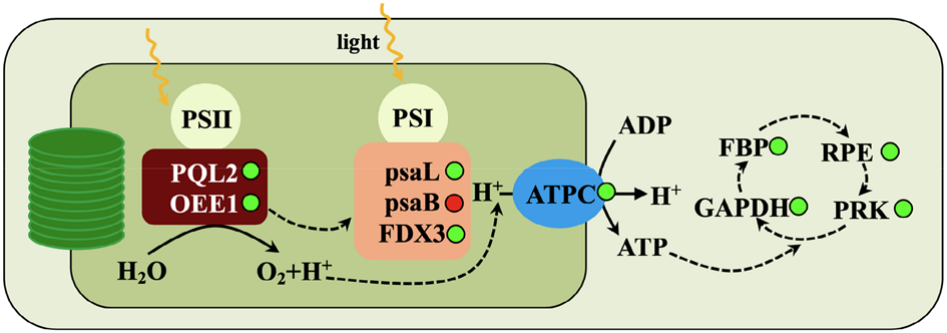
Schematic presentation of photosynthesis-associated DEGs after 400 mM NaCl treatment in the leaf epidermis of *A. marina*.

It has been reported that salt stress causes various effects on plant growth, especially on photosynthesis (*P*_n_)^20^. The present study also supports this conclusion, that is, the photosynthetic rate was significantly decreased under 400 mM NaCl treatment, implying that *P*_n_ was repressed in *A. marina* seedlings. The transcriptomic results of the upper epidermis further confirmed our hypothesis. Ten *P*_n_-related differentially expressed genes (DEGs) were identified and significantly expressed after 400 mM NaCl treatment (Figs. 6, 7 and Supplementary Table S1). Among them, five were down-regulated and involved in the light reaction. These genes that encode oxygen-evolving enhancer protein 1 (OEE1), photosynthetic NDH subunit of lumenal location 2 (PQL2), ferredoxin-3 (FDX3), and ATP synthase gamma chain (ATPC) (Figs. 6, 7 and Supplementary Table S1). OEE1 localizes to chloroplasts, as a subunit of OEE is necessary for plant photosystem II (PS II) assembly/stabilization^21^. It was previously suggested that OEE1 is important for the salinity tolerance of a mangrove plant *B. gymnorrhiza*^22^. PQL2 functions as a NAD(P)H dehydrogenase (NDH) subunit that is necessary for the chloroplast NDH complex in higher plants and was proven to be essential for the protective or adaptive mechanisms of plants during salt stress^23,24^. FDX was revealed to be a major electron donor that could transfer electrons in diverse redox-driven metabolic reactions and mediate the cyclic electron flow around PSI^25^. In this study, the transcript levels of *OEE1*, *PQL2* and *FDX* were markedly decreased, implying that 400 mM NaCl treatment could depress the *P*_n_ of *A. marina* by affecting the PS II complex and related electron transportation. *ATPC* was decreased under 400 mM NaCl treatment, suggesting that less ATP was available for other biological processes, such as regulating ribulose-1,5-bisphosphate. This was consistent with our proteomic results^2^. In addition, four-dark reaction correlated DEGs were identified (Figs. 6, 7 and Supplementary Table S1). As expected, they were all down-regulated, suggesting that the carbon fixation was also suppressed under 400 mM NaCl treatment. Similar results were also found in the proteomic results of this species^2^ and other mangrove plants^12,20^ with salt treatment.

### Effects of NaCl treatment on hormone signalling in *A. marina* seedlings

**Figure 8:**
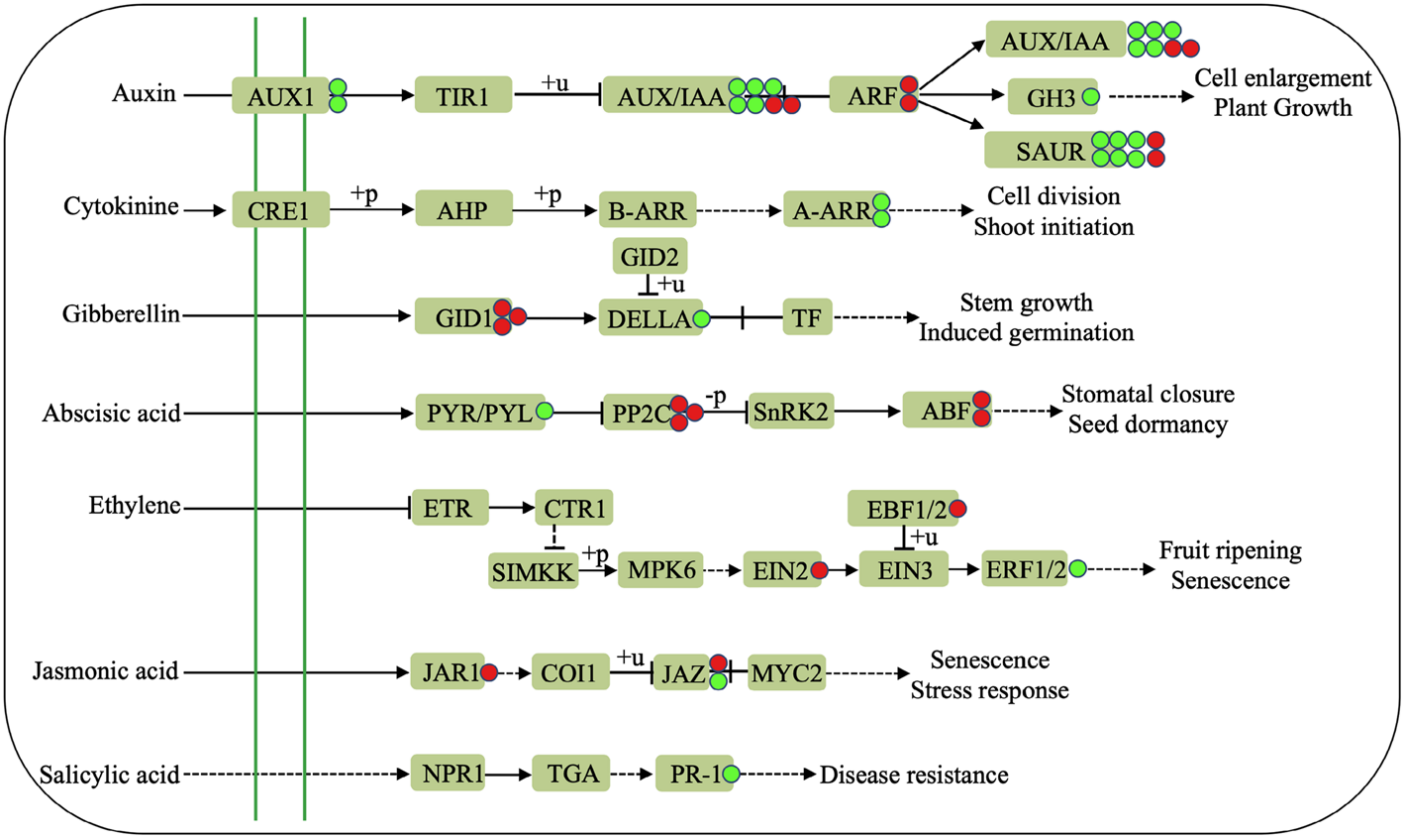
Schematic presentation of hormone signalling-associated DEGs after 400 mM NaCl treatment in the leaf epidermis of *A. marina*.

Phytohormones are a kind of small molecule organic substance that moves from the production site to the action site; at this site, phytohormones perform regulatory functions when plants face biotic and abiotic stresses^26^. Auxin (AUX) is an important plant hormone, and the transport process between cells is mainly affected by auxin export and input carriers. In this study, two genes encoding auxin influx carriers (AUX1) were markedly inhibited (Figs. 6, 8 and Supplementary Table S1), indicating that AUX was lower in the NaCl-treated leaf epidermis than in the control *A. marina* seedlings. It has been postulated that auxin/indole-3-acetic acid (AUX/IAA) proteins are repressors that do not target the TGTCTC auxin response element (AuxRE) directly; instead, they bind to AuxREs by dimerizing with auxin response factor (ARF) transcriptional activators when auxin concentrations are low, resulting in the repression of primary/early auxin response genes (grouped into three major groups: AUX/IAA, GH3, and SAUR gene families)^27^. Our results are consistent with this hypothesis, and sixteen genes were identified, which were mostly repressed (Figs. 6, 8 and Supplementary Table S1); this repression might be attributed to the lower AUX caused by the suppression of AUX1. In addition, we identified two *ARF3* genes in the AUX signal transduction pathway (Figs. 6, 8 and Supplementary Table S1). Tiwari et al.^27^ showed that *SlARF4* expression conferred *Solanum lycopersicum* with a greater capability to tolerate salinity. Kang et al.^28^ demonstrated that the overexpression of *IbARF5* from *Ipomoea batatas* in *Arabidopsis* strengthens the plant’s salt and drought tolerance. In this study, the up-regulation of ARF3 genes highlights the potential role of *A. marina* in response to saline habitats.

It is widely believed that cytokinins (CKs) are related to plant development. More recently, the roles of CKs in plant stress defence have become increasingly prominent. The AHK → AHP → ARR phosphorelay network was deemed to participate in CK signal transduction. In this study, we obtained two *ARRs*, *ARR9* and *ARR15*, that were restrained after *A. marina* exposure to salinity (Figs. 6, 8 and Supplementary Table S1). ARRs are generally divided into the following two categories: type-A and type-B ARRs. A study on *Arabidopsis* has demonstrated that the double mutant in two type-B ARRs, *arr1/arr12*, is more tolerant to salinity^29^. Similarly, in rice, *OsRR10* is repressed by salinity, and the *osrr9/osrr10* functional deletion mutant shows enhanced salt tolerance with higher photosynthetic efficiency and lower ion leakage and oxidative status^30^. Combined with a previous study^29,30^, we proposed that *ARR9* and *ARR15* might function as negative regulators for the tolerance of *A. marina* to salinity.

Four genes were involved in gibberellin (GA) signal transduction (Figs. 6, 8 and Supplementary Table S1). Studies have shown that the GID1 and DELLA proteins play important roles in plant growth regulation. GID1 is a gibberellin signal receptor, and DELLA protein is a key repressor of plant growth and development^31^. The induction of GID1B and inhibition of GAI1 in our current study imply that GA signalling functions in response to the salinity stress in *A. marina.*

The dual negative regulatory system PYR/PYL/RCAR⊣PP2C⊣SnRK2 is mainly recognized to regulate abscisic acid (ABA) signal transduction and its downstream response. In the present study, we also identified genes differentially expressed in the ABA signal transduction pathway (Figs. 6, 8 and Supplementary Table S1). Wang et al.^32^ analysed the expression of the *PP2C* gene family at the whole gene level, and six *StPP2C* genes were significantly up-regulated under high salt treatment. Similar expression patterns were also found in *Glycine max*^33^ and *Populus trichocarpa*^34^. The above results suggested that *PP2C* may be activated in the response process to salt treatment. Therefore, we speculated that 400 mM NaCl treatment could adjust the activity of SnRK2 by altering the expression of *PP2C* to ensure that plants could quickly recover from the stress response to a normal growth state, thus leading to the down-regulation of *PYL* expression in this study. In addition, ABA insensitive protein 5 (ABI5), a transcription factor of basic leucine zipper (bZIP), plays an important role in ABA signalling. As revealed in rice^35^ and maize^36^, ABI5 was induced under salt stress, and the growth of overexpressed transgenic plants was significantly inhibited. In Arabidopsis, the survival rate of *ABI5* loss-of function plants was significantly lower than that of CK plants^37^. Similarly, in this study, two genes encoding ABI5 were up-regulated under salt treatment, indicating that *ABI5* could act as a transcription factor to modulate the growth and salinity adaptability of *A. marina* seedlings.

Three DEGs were obtained in ethylene (ETH) signalling (Figs. 6, 8 and Supplementary Table S1). ETH insensitive 2 (EIN2) may be the central element of ETH signal transduction in plants, and is responsible for transmitting ETH signals from the endoplasmic reticulum to the nucleus. Under NaCl treatment, *ein2-1* and *ein2-5* mutants showed severe salt sensitivity in Arabidopsis, in which the outer epidermis exhibits backwards growth, rosettes are smaller and electrolyte permeability is higher. The introduction of CEND in *ein2-5* mutants can restore the salt tolerance phenotype and related physiological parameters^38^. The increased expression of *EIN2* after NaCl treatment, emphasizes its essential role in the salt tolerance of *A. marina*. In addition, a previous investigation revealed that EIN3-binding F-box protein 1 (EBF1) and EBF2 negatively regulate the stability of EIN3, another important factor in ETH signalling associated with salt tolerance in plants^39^. In this study, the up-regulated expression of EBF2 and the down-regulated expression of ETH-responsive transcription factor 1B (ERF1B) imply that appropriate ethylene signalling was involved in the response of *A. marina* to NaCl treatment.

In addition, DEGs involved in jasmonic acid (JA) and salicylic acid (SA) signal transduction pathways were also identified, such as genes encoding JA-amino synthetase (JAR1) and pathogenesis-related protein (PR) (Figs. 6, 8 and Supplementary Table S1). JA, as an important plant stress hormone, is crucial in regulating salt tolerance in plants. It was reported that the endogenous JA content was higher in salt-tolerant *Oryza sativa* cultivars than in salt-sensitive cultivars. Moreover, exogenous JA could improve the salt tolerance of rice^40^ and the resistance of *Glycine Max*^41^ to salt stress. JAR1 can catalyse JA to form JA-isoleucine (JA-Ile), the active form of JA, thereby triggering the JA signalling. PRs are involved in SA signalling, and it was demonstrated that the expression of rice PR2 and PR3 proteins were up-regulated with the prolongation of salt stress, while PR1a, PR5, PR8 and PR16 were down-regulated^42^. In the present study, *JAR1* and *PR6* were differentially expressed after NaCl treatment, suggesting that JA and SA signalling were involved in the salt tolerance of *A. marina*.

### Effects of NaCl treatment on sulfur metabolism of *A. marina* seedlings

**Figure 9:**
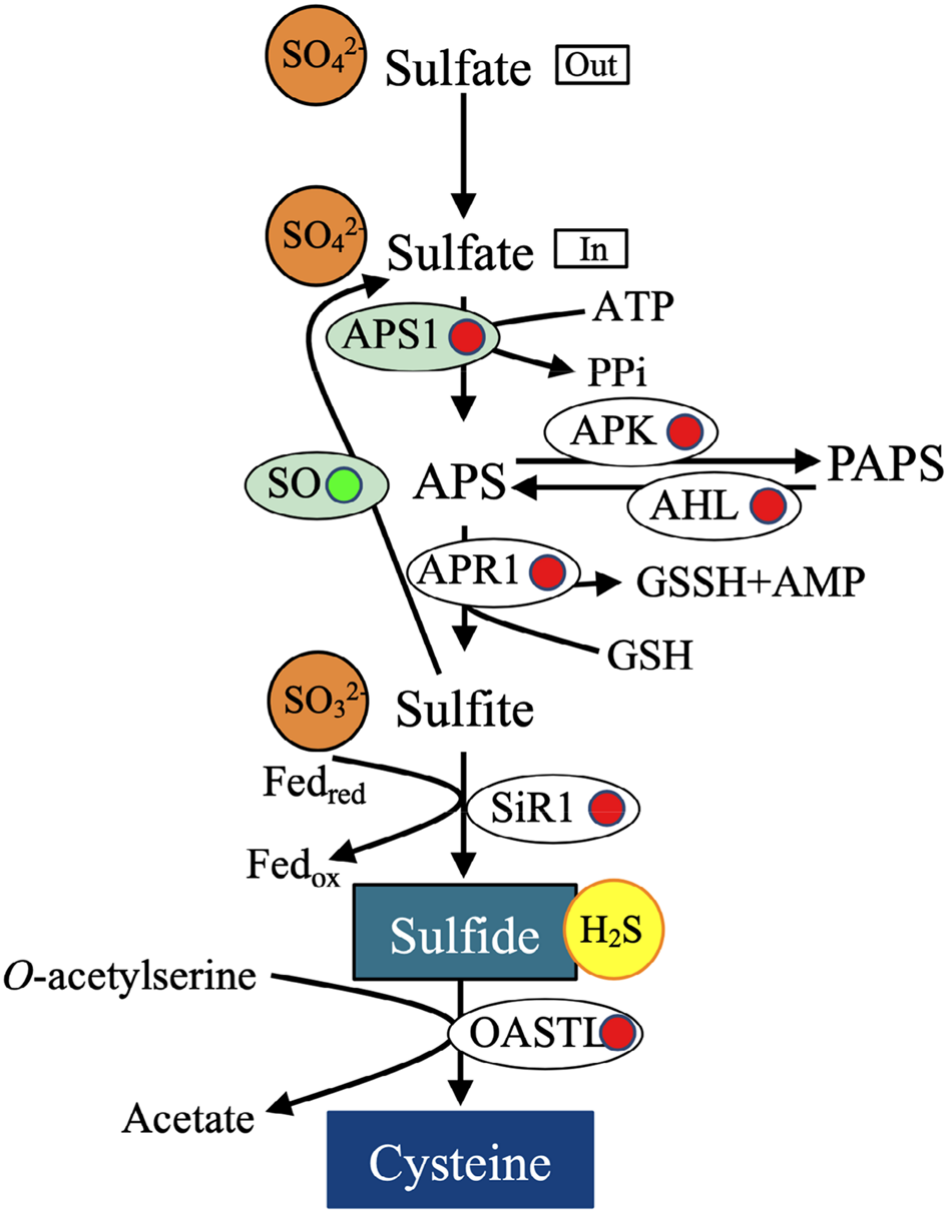
Schematic presentation of sulfur metabolism-associated DEGs after 400 mM NaCl treatment in the leaf epidermis of *A. marina*.

Sulfur-containing defensive substances (SDCs) play an essential role in plant tolerance to biotic and abiotic stress^43^. In this study, seven DEGs related to sulfur metabolism were identified and shown to be involved in the biosynthesis of hydrogen sulfide (H_2_S) and cysteine. H_2_S, as a new gas signalling molecule, is crucial because it plays a regulatory role in animals and plants^20^. Our previous study showed that H_2_S plays a protective role in the salt tolerance of rice^44^. In addition, physiological and proteomic results revealed that H_2_S plays roles in *K. obovata* coping with high salinity treatment by enhancing photosynthesis and energy metabolism^20^. Therefore, we inferred that H_2_S is involved in regulating the response process of *A. marina* to salinity treatment. Indeed, our hypothesis was verified by the significantly up-regulated expression of genes encoding ATP sulfurylase 1 (APS1), 5′ adenylylsulfate reductase 1 (APR1) and sulfite reductase 1 (SiR1) (Fig. 9 and Supplementary Table S1). In the process of sulfur metabolism, H_2_S and O-acetylserine (OAS) can synthesize cysteine under catalysis by the OAS (thiol) lyase (OASTL) enzyme. Inorganic sulfur can be incorporated into cysteine through this reaction and then converted into other sulfur compounds^45,46^. In this study, the expression pattern of OASTL was up-regulated, implying that NaCl treatment induced the cysteine biosynthesis in *A. marina* seedlings, which could provide more precursors for other sulfur-containing metabolites, and thus promote the adaptability of *A. marina* to salt.

## Conclusions

The effects of salinity treatment on the leaf epidermis of *A. marina* seedlings were unravelled by physiological techniques coupled with transcriptomic methods in this study. We found that 400 mM NaCl caused negative effects on the growth of *A. marina* seedlings. Further transcriptomic data revealed that hormone signal transduction and sulfur metabolism play essential roles in the response of *A. marina* to salt treatment. In addition, the depressed expression patterns of photosynthesis-related genes could explain the inhibition of photosynthesis. Overall, the present study will expand research on the salt tolerance mechanism of mangrove plants, and provide scientific guidance for the restoration and management of mangrove plantations.

## Materials and methods

### Plant culture conditions and treatments

Propagules of *A. marina* were collected from the Zhangjiang estuary mangrove national nature reserve in Fujian Province of China. Collection of mangrove propagules was permitted by the local administration and followed relevant institutional, national, and international guidelines and legislation. Voucher specimens were stored in the Herbarium of the School of Life Sciences, Xiamen University, with specimen code XYZ20180818^47^. Propagules with uniform size and free of bacteria and pests were planted randomly into culture basins filled with washed sand and irrigated with water. After germination, plants were cultured with whole Hoagland nutrient solution in a greenhouse with a relative humidity of 60–70%, an air temperature of 25–28 °C, and a 12 h/12 h (dark/light) cycle with a light intensity of 800–1000 μmol m^−2^ s^−1^ photosynthetically active radiation (PAR)^3^.

After two months of plant culture, seedlings of *A. marina* with consistent growth were randomly selected and divided into two groups. One group was set as the control (only Hoagland nutrient solution without extra NaCl addition), while another group was set as Hoagland nutrient solution containing 400 mM NaCl (400 mM NaCl treated group). Salt was added at a rate of 100 mM every three days until the required salinity was reached. The solution of each treatment was changed every three days with three replicates, and each replicate contained twenty individual plants^4^. After thirty days of treatment, some of the second pairs of *A. marina* seedling leaves were selected for biomass measurement and photosynthetic analysis, and others were used for the corresponding transcriptomic study.

### Obtaining measurements for the biomass and photosynthesis parameters

The biomass of *A. marina* leaves was measured using the weighting method^48^. Leaf areas were determined by a MicroTek ScanWizard EZ scanner and LA-S image analysis software as described in our previous study^49^. In addition, the photosynthesis parameters of the second pair of fully developed leaves were measured with a portable photosynthesis system (Li-6400, Li-Cor, Lincoln, NE, USA)^3^.

### RNA extraction and data processing

The epidermises of 0 mM NaCl (CK) and 400 mM NaCl (NaCl) treated *A. marina* seedlings were thoroughly ground in liquid nitrogen. Total RNA was obtained with the Bioteke RNA separation kit (Bioteke Corporation, China) and checked by an Agilent 2100 bioanalyzer. After RNA quality assessment, the total RNA was used for cDNA library construction^49^. Briefly, mRNA was purified with poly-T oligo-attached magnetic beads and fragmented by divalent cations in fragmentation buffer. The synthesis of the first strand of cDNA was performed in the M-MuLV reverse transcriptase system with the above obtained mRNA as templates and random oligonucleotides as primers. Following degradation of the RNA strand by RNase H, the second strand of cDNA was synthesized in the DNA polymerase I system by using dNTPs as raw materials. After end repair, addition of A tail and adaptor of purified cDNA, AMPure XP system were utilized to screen for approximately 370 to 420 bp of cDNA. PCR amplification was performed, and the AMPure XP system was used again to purify the PCR product. Libraries were ultimately obtained and assessed by an Agilent 2100 bioanalyzer. The clusters were generated by utilizing a cBot Cluster Generation System based on the manufacturer’s instructions. Next, the library preparations were sequenced by the Illumina NovaSeq 6000 platform to produce 150 bp paired-end reads^13,49^. The relevant sequenced raw data were deposited to Sequence Read Archive (SRA) with the accession number SRR22993527.

To ensure the quality and reliability of data analysis, the raw data were filtered by removing reads with low quality (Q_phred_ ≤ 20), adapters and poly-N sequences. The Q20, Q30 and GC contents were also obtained. After quality control, clean reads were referenced to the *A. marina* genome (GSA database with accession number CRA004669)^50^ and utilized for the subsequent analysis. In addition, FPKM values and DESeq2 were used for differential gene expression analysis^51,52^. Genes with the threshold of the adjusted *p* value (*P*_adj_) < 0.05 and |log2FoldChange| > 1 were defined as differentially expressed genes (DEGs). GOseq was utilized to perform Gene Ontology (GO) enrichment analysis of the RNA-seq transcriptome data with *p* < 0.01^53^. Kyoto Encyclopedia of Genes and Genomes (KEGG) enrichment analysis was performed according to the methods of Mao et al.^54^ using the KEGG Ontology (KO)-based annotation system with *p* < 0.001.

### Statistical analysis

Statistical analyses were performed with one-way ANOVA in SPSS software (Version 20) followed by Duncan’s multiple range test under *p* < 0.05^4^. The Venn diagram, hierarchical clustering and the volcanic plot were analysed on the OmicShare online platform (https://www.omicshare.com/). The schematic presentations of related pathways were plotted based on KEGG Mapping (https://www.kegg.jp/kegg/kegg1b.html) and the reported literature^55–57^. Figures were drawn with GraphPad Prism (Version 9.0.2), and all data shown in the figures are represented as the means ± SEs.

## Supplementary Information

Supplementary Table S1.

## Data Availability

The data supporting this work is publicly available from NCBI SRA (Sequence Read Archive) with the accession number SRR22993527.
