# Supplementary material for: RNA-sequencing transcriptome analysis of Avicennia marina (Forsk.) Vierh. leaf epidermis defines tissue-specific transcriptional response to salinity treatment
Source: Sci Rep. 2023 May 10;13:7614. doi: 10.1038/s41598-023-34095-x (PMC10172313; doi:10.1038/s41598-023-34095-x)
Supplement: Supplementary file 1 — Supplementary Table S1. [file 41598_2023_34095_MOESM1_ESM.docx]

Supplementary Table S1: DEGs identified in the transcriptome data of *A. marina* seedlings after 400 mM NaCl treatment.

| Functional Category | Gene ID | RPKM (NaCl) | RPKM (CK) | Log2FoldChange | padj | Gene Description |
| --- | --- | --- | --- | --- | --- | --- |
| Photosynthesis | | | | | | |
|  | novel.1805 | 397.01 | 109.80 | 1.86 | 4.25E-04 | Photosystem I P700 chlorophyll a apoprotein A2 (psaB) |
|  | Am26050 | 4103.21 | 10723.47 | -1.39 | 1.11E-03 | Oxygen-evolving enhancer protein 1 (OEE1) |
|  | Am15771 | 56.86 | 201.39 | -1.83 | 1.66E-03 | Ferredoxin-3 (FDX3) |
|  | Am07764 | 4127.65 | 9149.68 | -1.15 | 1.87E-02 | Photosystem I reaction center subunit XI (psaL) |
|  | Am07346 | 449.35 | 932.91 | -1.05 | 3.13E-02 | ATP synthase gamma chain (ATPC) |
|  | Am16405 | 254.36 | 774.97 | -1.61 | 3.65E-02 | Photosynthetic NDH subunit of lumenal location 2 (PQL2) |
|  | Am13000 | 1823.98 | 4323.55 | -1.25 | 2.00E-03 | Glyceraldehyde 3-phosphate dehydrogenase B (GAPDH) |
|  | Am23303 | 123.33 | 318.82 | -1.37 | 2.29E-03 | Phosphoribulokinase (PRK) |
|  | Am11796 | 1272.56 | 2854.03 | -1.16 | 4.40E-03 | Fructose-1,6-bisphosphatase (FBP) |
|  | Am30009 | 11.47 | 51.36 | -2.16 | 3.95E-02 | Ribulose-phosphate 3-epimerase (RPE) |
| Plant hormone signaling | | | | | | |
|  | Am08112 | 130.82 | 476.10 | -1.86 | 4.43E-06 | Auxin transporter-like protein 2 (AUX1) |
|  | Am11780 | 794.66 | 1722.89 | -1.12 | 3.92E-05 | Auxin transporter-like protein 2 (AUX1) |
|  | Am16010 | 1742.41 | 4183.10 | -1.26 | 3.39E-05 | Auxin-induced protein 22D (AUX22D) |
|  | Am15210 | 2341.46 | 5040.58 | -1.11 | 1.08E-03 | Auxin-induced protein 22B (AUX22B) |
|  | Am02219 | 771.23 | 322.95 | 1.26 | 1.18E-04 | Auxin-responsive protein IAA11 (IAA11) |
|  | Am22594 | 0.00 | 26.27 | -7.10 | 8.43E-04 | Auxin-responsive protein IAA16 (IAA16) |
|  | Am27599 | 328.20 | 1177.19 | -1.84 | 2.68E-03 | Auxin-induced protein IAA6 (IAA6) |
|  | Am15209 | 1358.59 | 2792.14 | -1.04 | 3.41E-03 | Auxin-responsive protein IAA14 (IAA14) |
|  | Am27642 | 88.69 | 31.64 | 1.48 | 1.77E-02 | Auxin-responsive protein IAA26 (IAA26) |
|  | Am28863 | 977.93 | 107.57 | 3.19 | 3.04E-17 | Auxin response factor 3 (ARF3) |
|  | Am22515 | 205.57 | 70.16 | 1.55 | 1.70E-03 | Auxin response factor 3 (ARF3) |
|  | Am07562 | 46.84 | 663.82 | -3.83 | 1.01E-10 | Indole-3-acetic acid-amido synthetase GH3.6 (GH3.6) |
|  | Am13234 | 186.75 | 24.04 | 2.94 | 6.48E-07 | Auxin responsive protein (SAUR) |
|  | Am11696 | 367.10 | 1155.38 | -1.65 | 2.07E-06 | Auxin-responsive protein SAUR50 (SAUR50) |
|  | Am09889 | 1126.22 | 4037.43 | -1.84 | 7.07E-06 | Auxin-responsive protein SAUR50 (SAUR50) |
|  | novel.998 | 180.46 | 645.68 | -1.84 | 1.07E-04 | Auxin-responsive protein SAUR20 (SAUR20) |
|  | Am05364 | 32.85 | 130.36 | -1.99 | 4.39E-02 | Auxin-responsive protein SAUR24 (SAUR24) |
|  | Am09886 | 379.11 | 1034.37 | -1.45 | 1.51E-03 | Auxin-responsive protein SAUR24 (SAUR24) |
|  | Am23092 | 122.26 | 31.04 | 1.98 | 3.73E-03 | Auxin-responsive protein SAUR36 (SAUR36) |
|  | Am17286 | 7.11 | 64.11 | -3.17 | 1.92E-02 | Auxin-responsive protein SAUR50 (SAUR50) |
|  | Am01031 | 50.61 | 204.37 | -2.02 | 4.25E-05 | Two-component response regulator ARR9 (ARR9) |
|  | Am27007 | 31.54 | 131.03 | -2.05 | 5.36E-03 | Two-component response regulator ARR15 (ARR15) |
|  | Am07926 | 1904.47 | 511.07 | 1.90 | 7.99E-17 | Gibberellin receptor GID1B (GID1B) |
|  | Am00720 | 212.00 | 9.35 | 4.48 | 2.19E-11 | Gibberellin receptor GID1B (GID1B) |
|  | Am02047 | 175.70 | 33.87 | 2.38 | 4.25E-05 | Gibberellin receptor GID1B (GID1B) |
|  | Am19295 | 190.08 | 527.84 | -1.47 | 1.02E-05 | DELLA protein GAI1 (GAI1) |
|  | Am08071 | 41.72 | 307.12 | -2.88 | 9.75E-06 | Abscisic acid receptor PYL4 (PYL4) |
|  | Am21605 | 184.34 | 10.84 | 4.10 | 5.40E-05 | Probable protein phosphatase 2C 51 (PP2C51) |
|  | Am12558 | 170.01 | 50.67 | 1.74 | 3.99E-03 | Protein phosphatase 2C 16 (PP2C16) |
|  | Am01402 | 2727.55 | 1113.47 | 1.29 | 7.09E-03 | Probable protein phosphatase 2C 6 (PP2C6) |
|  | Am13380 | 98.78 | 20.22 | 2.30 | 6.24E-03 | Abscisic acid insensitive 5-like protein 5 (AIF5) |
|  | Am12792 | 335.01 | 165.61 | 1.01 | 9.50E-03 | Abscisic acid insensitive 5-like protein 2 (AIF5) |
|  | Am11884 | 483.52 | 217.59 | 1.15 | 3.78E-04 | Ethylene-insensitive protein 2 (EIN2) |
|  | Am04207 | 1909.27 | 796.39 | 1.26 | 6.29E-05 | EIN3-binding F-box protein 2 (EBF2) |
|  | Am28087 | 35.55 | 434.48 | -3.61 | 5.75E-12 | Ethylene-responsive transcription factor 1B (ERF1B) |
|  | Am11045 | 10195.67 | 3513.96 | 1.54 | 1.67E-05 | Jasmonic acid-amido synthetase JAR1 (JAR1) |
|  | Am27139 | 611.51 | 1264.07 | -1.05 | 2.15E-04 | Protein TIFY 6B (TIFY6B) |
|  | Am05746 | 394.47 | 196.42 | 1.01 | 8.91E-04 | Protein TIFY 10B (TIFY10B) |
|  | Am09678 | 2.51 | 101.77 | -5.36 | 5.13E-05 | Pathogenesis-related leaf protein 6 (PR1B1) |
| Sulfur metabolism | | | | | | |
|  | Am13969 | 4149.67 | 770.84 | 2.43 | 3.86E-07 | ATP sulfurylase 1 (APS1) |
|  | Am02308 | 1311.92 | 547.51 | 1.26 | 8.59E-05 | Sulfite reductase 1 (SiR1) |
|  | Am21991 | 652.51 | 260.57 | 1.32 | 1.99E-03 | PAP-specific phosphatase HAL2-like (AHL) |
|  | Am24739 | 347.24 | 85.03 | 2.04 | 7.18E-03 | Cysteine synthase (OASTL) |
|  | Am07350 | 5541.88 | 2729.11 | 1.02 | 8.52E-03 | 5' adenylylsulfate reductase 1 (APR1) |
|  | Am15405 | 0.00 | 11.32 | -5.89 | 2.49E-02 | Sulfite oxidase (SO) |
|  | Am29381 | 8972.03 | 4467.16 | 1.01 | 3.82E-02 | Adenylyl-sulfate kinase (APK) |
